# Supplementary material for: Learning curve and surgical outcome of robotic assisted colorectal surgery with ERAS program
Source: Sci Rep. 2022 Nov 29;12:20566. doi: 10.1038/s41598-022-24665-w (PMC9709162; doi:10.1038/s41598-022-24665-w)
Supplement: Supplementary file 1 — Supplementary Information 1. [file 41598_2022_24665_MOESM1_ESM.zip › LSC Raw data-Ñ[▒K/115/O_ñw╝╨░O▒Kñσ.pdf]

正式摘要(電子簽章：000000209200880)

W52 / W52

臺中榮民總醫院

Operation Note

[redacted]

- 手術日期：2021/01/28
- 手術醫師：蔣鋒帆，Fellow 江世偉，CR 楊曙榮，R 陳凱祥
- Anesthesia：GE
- Position：Supine
- Aseptic agents：Soap，Betadine Alcohol，Betadine Aqua

Diagnosis

- Pre-operative diagnosis : Malignant tumor of T-colon
- Post-operative diagnosis : DITTOs/p laparoscopic right hemicolectomy

Surgical Indication

- Removal of tumor

Operation

- Laparoscopic right colectomy and anastomosis
  Side to side anastomsis with GIA 80/3.8mmx2
  Surgiwrap +

Finding

- 1. One polypoid tumor 6x5cm in size over middle T-colon
  2. No liver metastasis and no peritoneal seeding
  3. Side to side anastomosis with GIA 80/3.8 with two fires.
  4. Ligation of ileocolic, right colic, right branch of middle colic and middle colic vessels.
  5. One 12mm penros drain to anastomosis
  6. Surgiwrap:+
  7. Severe adhision around A colon, T-colon, omentum and Gall bladder
  8. Redundent T and S noted

Procedure

- Under general anesthesia, the patient was placed in the supine position with legs in adducted position. The abdomen was prepped and draped in standard sterile fashion. A small vertical incision was made below the umbilicus and a 12mm trocar was inserted into the peritoneal cavity by open method. Pneumoperitoneum was established with CO2 inflation. Laparoscope was inserted and then a 12mm trocar were inserted over LUQ of abdomen. Two 5mm trocars were inserted over RLQ and LLQ of abdomen. The liver, omentum and peritoneum were inspected for evidence of metastatic disease. No metastatic disease was noted. The peritoneum overlying the mesentery was then scored with electrocautery and the ileocolic artery was identified, ligated with endoclips, and transected. The remaining vessels to the cecum were also divided and ligated. Using Harmonica, omentum was freed from the transverse colon and lesser sac was entered. The colon was freed from its peritoneal attachments along the line of Toldt from the cecum to the hepatic flexure. Hemostasis was checked in the operative field. Cecum, ascending colon hepatic flexure and T-colon were mobilized and then laparoscope was removed. One midline incision was made at peri-umbilical region. The ascending colon was delivered into the incision and points of transection were selected proximally and distally. Side to side anastomosis was performed with GIA 80/3.8. Interrupted 3-0 silk Lambert sutures were used to re-inforcement at outer layer. The anastomosis was checked and found to be intact and widely patent. One 12mm penrose was placed at sub-hepatic region. The fascia was closed with interrupted sutures of No.1 Vicryl. The fascia of the 12 mm trochar wound

- was closed with 1-0 vicryl. The skin was closed with 3-0 Nylon. The patient tolerated the procedure well and was brought to the SICU for poor lung function.
- Estimated blood loss: minimum
- Blood transfusion FFP 6 unit.
- 器械數量: 正確
- 紗布數量: 正確

Image

- PAINT
  - abdomen

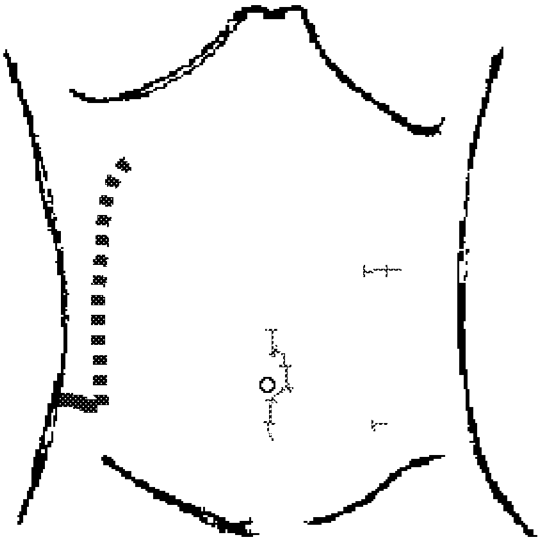

  - colon & rectum

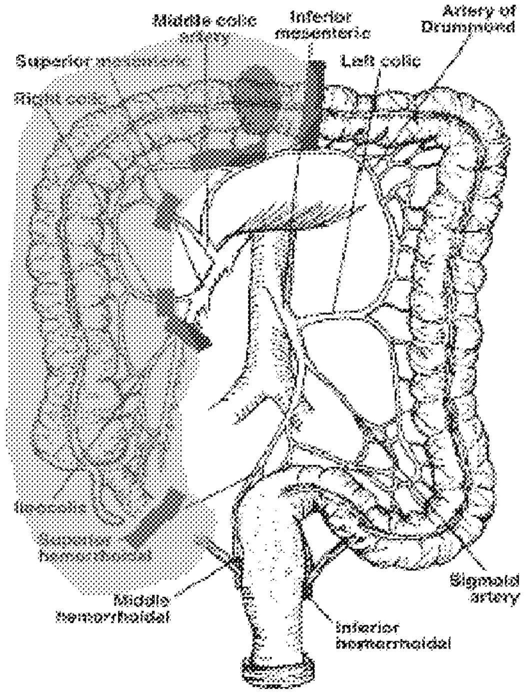

主任 2062F蔣鋒帆 / 醫師 2292G楊曙榮

正式摘要(電子簽章：**000000209200880)**

W52 / W52

doctorRank:B
